# Protocol to reconstitute translationally arrested heat shock mRNPs and condensates *in vitro*

**DOI:** 10.1016/j.xpro.2024.103275

**Published:** 2024-08-21

**Authors:** Christine Desroches Altamirano, Simon Alberti

**Affiliations:** 1 Biotechnology Center (BIOTEC), Center for Molecular and Cellular Bioengineering, Technische Universität Dresden, Tatzberg 47/49, 01307 Dresden, Germany

**Keywords:** Cell Biology, Protein Biochemistry, Protein expression and purification

## Abstract

Heat shock (HS) coincides with the assembly of translationally arrested heat shock messenger ribonucleoprotein particles (HS-mRNPs) and condensates. Here, we present a protocol to reconstitute HS-mRNPs and HS condensates with eIF4G, eIF4E, Pab1p, and mRNA *in vitro*. In addition, we describe the necessary steps to measure the effect of HS-mRNPs and HS condensates on translation in yeast extracts. The protocol can be modified to study mRNPs and condensates assembled with other proteins and to study translation in extracts prepared from different cells.

For complete details on the use and execution of this protocol, please refer to Desroches Altamirano et al.^1^

## Highlights

• Instructions for the purification of eIF4G, Pab1p, and eIF4E
• Steps for reconstituting heat shock mRNPs and condensates *in vitro*
• Steps for measuring the effect of reconstituted mRNPs and condensates on translation using yeast extracts

## Abstract (editor highlights)

Publisher’s note: Undertaking any experimental protocol requires adherence to local institutional guidelines for laboratory safety and ethics.

## Graphical abstract

**Figure:**
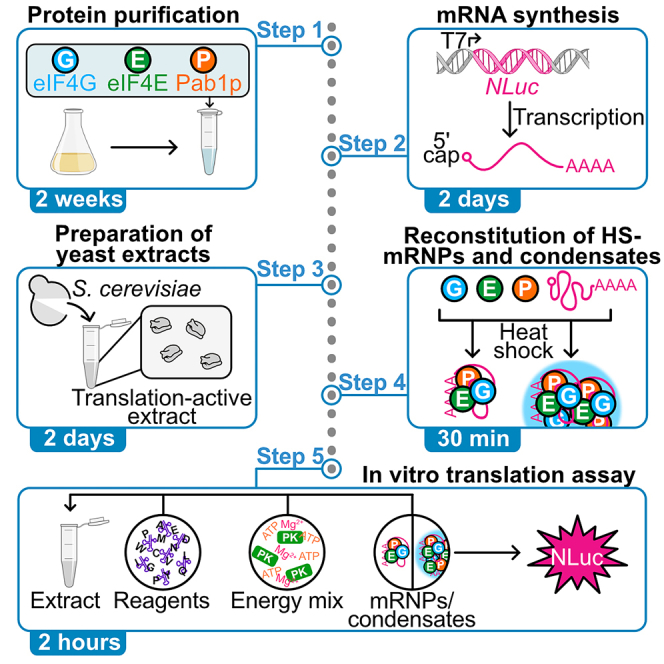

## Before you begin

In this protocol, we describe the procedure to reconstitute messenger ribonucleoprotein particles (mRNPs) and condensates with eIF4G, eIF4E and Pab1p. Heating of reconstituted mRNPs and condensates results in the assembly of heat shock (HS)-mRNPs and condensates. HS-mRNPs and HS-condensates exhibit strengthened interactions and become dynamically arrested. Crucially, only the addition of HS-mRNPs and HS-condensates repress translation in extracts prepared from yeast cells.^1^

In this protocol, we describe the steps required to reconstitute eIF4G-eIF4E-Pab1p mRNPs and condensates with mRNA encoding Nanoluciferase (NLuc) which is used as a reporter for translation in yeast extracts. First, we describe the steps for the purification of eIF4G, eIF4E and Pab1p and the synthesis of mRNA using a commercially available *in vitro* transcription kit. The plasmids utilized for protein purification and mRNA synthesis are deposited and available at Addgene. Recombinant eIF4G and Pab1p are expressed in *Spodoptera frugiperda* Sf21 (Sf9) with a baculovirus expression system,^2^ while recombinant eIF4E is expressed in *E. coli*. Next, we describe the steps required to assemble mRNPs and condensates *in vitro* with purified proteins and *in vitro* transcribed *NLuc* mRNA. In addition, we present a protocol for the preparation of cell-free extracts from yeast cells. Finally, we outline the steps required to establish *in vitro* translation assays using yeast extracts and reconstituted mRNPs and condensates.

## Key resources table

**Table:** 

REAGENT or RESOURCE	SOURCE	IDENTIFIER
**Bacterial and virus strains**		

*Spodoptera frugiperda* cell line IPLB-Sf-21-AE (Sf9) cells	Expression Systems, Davis, CA, USA	Cat #94-001F
BL21-AI One Shot chemically competent *E. coli*	Thermo Fisher Scientific	Cat #607003

**Chemicals, peptides, and recombinant proteins**		

Tris base	Merck	Cat #TRIS-RO
Potassium chloride (KCl)	Carl Roth	Cat #6781.1
EDTA, disodium salt, dihydrate	Merck	Cat #324503
DTT	Thermo Fisher Scientific	Cat #R0861
HEPES	Carl Roth	Cat #6763.3
Bond-Breaker TCEP	Thermo Fisher Scientific	Cat #77720
Potassium acetate	Merck	Cat #P1190-100G
Magnesium acetate	Sigma-Aldrich	Cat #M0631
D-mannitol	Sigma-Aldrich	Cat #M4125-10MG
Adenosine 5′-triphosphate disodium salt hydrate	Thermo Fisher Scientific	Cat #J61125.14
Guanosine 5′-triphosphate, sodium salt	Jena Bioscience	Cat #NU-1012
Creatine phosphate	Sigma-Aldrich	Cat #10621714001
Creatine phosphokinase	Sigma-Aldrich	Cat #C3755
Amino acid mixture (complete)	Promega	Cat #L4461
Yeast tRNA	Thermo Fisher Scientific	Cat# AM7119
RNaseOUT	Invitrogen	Cat #10777019
Glycerol	Carl Roth	Cat #3783.1
ESF AF insect cell culture medium	Expression Systems, Davis, CA, USA	Cat #99-300-01
Penicillin-streptomycin (10,000 U/mL)	Gibco	Cat #15140122
Fetal bovine serum (FBS)	Gibco	Cat #15353681
Escort IV transfection reagent	Sigma-Aldrich	Cat #L3287
SbfI-HF	NEB	Cat #R3642-S
Benzonase	Merck	Cat #E1014-5KU
cOmplete, EDTA-free protease inhibitor cocktail	Roche	Cat #05056489001
cOmplete protease inhibitor cocktail	Roche	Cat #11836145001
HRV 3C protease	Merck	SAE0045-1MG
L(+)-arabinose	Carl Roth	Cat #5118.2
7-Methylguanosine 5′-triphosphate (m^7^GTP) sodium salt	Sigma-Aldrich	Cat #M6133-5MG
5× Phusion HF buffer	Thermo Fisher Scientific	Cat #F-518
Phusion high-fidelity DNA polymerase	Thermo Fisher Scientific	Cat #F-518
dNTP mix (10 mM each)	Thermo Fisher Scientific	Cat #R0191
Aminoallyl-UTP-ATTO-488	Jena Bioscience	Cat #NU-821-488
2× RNA gel loading dye	Thermo Fisher Scientific	Cat #R0641
RiboRuler high range RNA ladder	Thermo Fisher Scientific	Cat #SM1821
Micrococcal nuclease	NEB	Cat #M0247S
Calcium chloride (CaCl_2_)	Carl Roth	Cat #CN93.1
EGTA	Carl Roth	Cat #3054.1

**Critical commercial assays**		

Monarch PCR & DNA cleanup kit	NEB	Cat #T1030L
mMESSAGE mMACHINE T7 transcription kit	Thermo Fisher Scientific	Cat# AM1344
Poly(A) tailing kit	Thermo Fisher Scientific	Cat #AM1350
Nano-Glo luciferase assay	Promega	Cat #N1110

**Experimental models: Organisms/strains**		

W303 ADE+	Gift, Zachariae lab	N/A

**Oligonucleotides**		

CDA1: CCTCTTCGCTATTACGC	Desroches Altamirano et al.^1^	N/A
CDA2: AGTTAGCTCACTCATTAGGC	Desroches Altamirano et al.^1^	N/A

**Recombinant DNA**		

DefBac	Lemaitre et al.^2^	N/A
Plasmid: pOCC102-MBP-eIF4G	Desroches Altamirano et al.^1^	Addgene #219971
Plasmid: pOCC102-MBP-Pab1	Desroches Altamirano et al.^1^	Addgene #219979
Plasmid: pOCC22-MBP-eIF4E	Desroches Altamirano et al.^1^	Addgene #219978
Plasmid: pUC57-T7-PAB1-NLuc	Desroches Altamirano et al.^1^	Addgene #219984

**Software and algorithms**		

Codon Optimization Tool	IDT	https://eu.idtdna.com/CodonOpt
Fiji (v2.0.0)	NIH	https://fiji.sc/
R (v 3.6.1)/RStudio (v1.4)	R Core Team	https://www.r-project.org/https://www.rstudio.com/
SparkControl v3.1	Tecan Life Sciences	N/A

**Other**		

Millex-GP PES filter, 0.2 μm, PES 33 mm	Merck	Cat #SLGP033NS
Countess cell counting chamber slides	Thermo Fisher Scientific	Cat #C10228
Countess II automated cell counter	Thermo Fisher Scientific	Cat #AMQAF1000
Breathe-Easy sealing membranes	Sigma-Aldrich	Cat #Z380059-1PAK
JA-25.50 fixed-angle rotor	Beckman Coulter	Cat #369660
Beckman Coulter Avanti J-25 high speed centrifuge	Beckman Coulter	Cat #369001
LM10 pneumatic microfluidizer processor	Microfluidizer	N/A
Amylose resin	NEB	Cat #E8021L
Econo-Pac chromatography column	Bio-Rad	Cat #7321010
Amicon Ultra centrifugal filters	Merck Millipore	Cat #UFC903024
Syringe filter, PES, 0.22 μm	Merck	Cat #10163282
HiLoad 16/600 Superdex 200 pg column	Cytiva	Cat #GE28-9893-35
ÄKTA pure protein purification system 25 M	Cytiva	Cat #29018226
Immobilized γ-aminophenyl-m^7^GTP (C10-spacer)	Jena Bioscience	Cat #AC-155L
Superdex 200 increase 10/300 GL column	Cytiva	Cat #GE28-9909-44
Centrifuge 5424 R	Eppendorf	Cat # 5424000018
Acid-washed glass beads (425–600 μm)	Sigma-Aldrich	Cat #G8772-10G
Laboratory Mixer Mill MM 400	Retsch	Cat #RET_12043
Zeba spin desalting columns, 7 K MWCO, 10 mL	Thermo Fisher Scientific	Cat #89894
Eppendorf ThermoMixer C	Eppendorf	Cat #5382000015
SafeSeal reaction tube, 1.5 mL, low protein-binding	Sarstedt	Cat #72.706.600
Prometheus nanoDSF grade high sensitivity capillaries	NanoTemper Technologies	Cat #PR-C006
Prometheus Panta	NanoTemper Technologies	Cat #PR003
CellCarrier Ultra ULA-coated 384-well microplate	Revvity Health Sciences Inc.	Cat #50-210-5656
384-well low volume white round bottom polystyrene NBS microplate	Corning	Cat #4513
The Spark multimode microplate reader	Tecan Life Sciences	Cat #30086376

## Materials and equipment

***Note:*** All buffers detailed below should be filtered through a Millex-GP PES Filter (0.22 μm) prior to use.

**Table:** Buffer A

Reagent	Concentration
Tris	50 mM
KCl	1 M
EDTA	2 mM
DTT	1 mM

The pH of the buffer with all components is adjusted to pH 7.5 with HCl. DTT is added directly before use and once added, the buffer is stable for 1 day at ∼23°C.

**Table:** Buffer B

Reagent	Concentration
Tris	50 mM
KCl	500 mM
EDTA	2 mM
DTT	1 mM

The pH of the buffer with all components is adjusted to pH 7.5 with HCl. DTT is added directly before use and once added, the buffer is stable for 1 day at ∼23°C.

**Table:** Buffer E

Reagent	Concentration
HEPES	50 mM
KCl	100 mM
DTT	1 mM

The pH of the buffer with all components is adjusted to pH 7.5 with KOH. DTT is added directly before use and once added, the buffer is stable for 1 day at ∼23°C.

**Table:** Buffer G

Reagent	Concentration
PIPES	20 mM
KCl	85 mM
TCEP	1 mM

The pH of the buffer with PIPES and KCl is adjusted to pH 7.0 with KOH. Buffer without TCEP is stable for > 2 years. TCEP is added directly before use and once added, the buffer is stable for 1 day at RT.

***Note:*** Make sure to add pH-neutralized TCEP, such as Bond-Breaker TCEP.

**Table:** Buffer Y/M

Reagent	Concentration
HEPES	30 mM
Potassium acetate	100 mM
Magnesium acetate	3 mM
Mannitol	8.5%
DTT	1 mM

Prior to the addition of Mannitol, adjust the pH of the buffer to pH 7.5 with KOH. The buffer is stable for 3 months at 4°C. DTT is added directly before use and once added, the buffer is stable for 1 day at 4°C.

**Table:** Buffer Y

Reagent	Concentration
HEPES	30 mM
Potassium acetate	100 mM
Magnesium acetate	3 mM
DTT	1 mM

Prior to the addition of Mannitol, adjust the pH of the buffer to pH 7.5 with KOH. The buffer is stable for 6 months at 4°C. DTT is added directly before use and once added, the buffer is stable for 1 day at 4°C.

**Table:** *In vitro* translation (IVT) reaction mix

Reagent	Stock concentration	Final concentration	Volume (μL)
Nuclease free water	N/A	N/A	38
HEPES	0.5 M	30 mM	120
ATP (pH neutralized)	100 mM	8 mM	160
GTP	100 mM	0.8 mM	16
Creatine phosphate	0.5 M	30 mM	120
Creatine phosphokinase (CPK)	10 U/μL	1.2 U/μL	240
Magnesium acetate	100 mM	20 mM	400
Potassium acetate	1 M	300 mM	600
Amino acids	1 mM	20 μM	40
tRNA	1 mM	120 μM	240
RNaseOUT	40 U/μL	0.4 U/μL	20
DTT	1 M	3 mM	6
Total	N/A	N/A	2000

Place ATP, GTP, creatine phosphate, CPK, amino acids, tRNA and RNaseOUT on ice. Prepare the IVT reaction mix on ice and store 100–200 μL aliquots in 1.5 mL tubes at −80°C.

∗ Prepare CPK stocks as detailed below.

***Note:*** Avoid thawing and freezing of the reaction mix. The activity of CPK in the reaction mix decreases when stored for longer than 3 months at −80°C.

**Table:** Preparation of 10 U/μL CPK

Reagent	Stock concentration	Final concentration	Amount
Nuclease free water	-	-	1.5 mL
HEPES pH 7.6	0.5 M	10 mM	70 μL
Potassium acetate pH 8.0	1 M	50 mM	175 μL
Glycerol	99%	50%	1.76 mL
Creatine phosphokinase	-	10 U/mL	110 mg (35,000 U)

Store at −80°C for up to 1 year.

## Step-by-step method details

### Purification of eIF4G and Pab1p

**Timing: 2 weeks**

**Figure 1:**
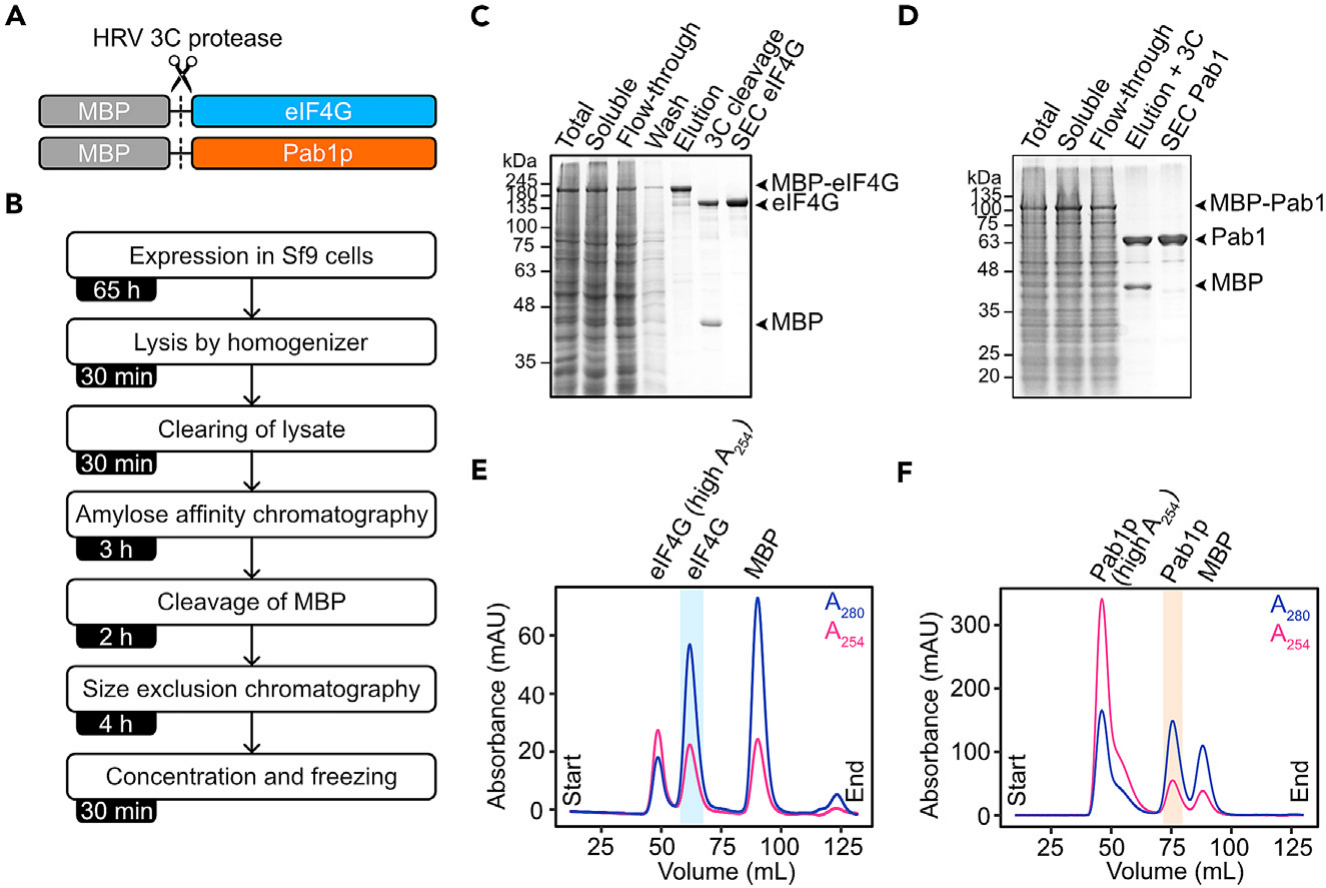
Purification of eIF4G and Pab1p (A) Schematic of MBP-eIF4G and MBP-Pab1p fusion protein constructs. The MBP is cleaved with HRV 3C protease. (B) Steps for the purification of eIF4G and Pab1p. (C) Coomassie stained 10% acrylamide gel of the purification steps of eIF4G. (D) Coomassie stained 10% acrylamide gel of the purification steps of Pab1p. (E) SEC chromatogram of eIF4G purification. Absorbance at 280 and 254 nm (A_280_ and A_254_ respectively) are shown. The elution peak with purified eIF4G is indicated. (F) SEC chromatogram of Pab1p purification. A_280_ and A_254_ are shown. The elution peak with purified Pab1p is indicated.

eIF4G and Pab1p are recombinantly expressed and purified from baculovirus-infected Sf9 cells (Figures 1A and 1B). eIF4G and Pab1p are expressed as fusion constructs with maltose binding protein (MBP) placed at the N-terminus. Fusion MBP-eIF4G and MBP-Pab1p are captured using amylose resin and further purified using size exclusion chromatography (SEC) (Figures 1C and 1D).

1. Prepare baculovirus stocks as previously described in Lemaitre et al. 2019.^2^
  a. Prepare transfection mix.
    **Table:** Transfection mix

Reagent	Volume (μL)
ESF AF Insect Cell Culture Medium	800
Escort IV transfection reagent	12
SbfI-digested DefBac DNA (0.2–0.4 μg/μL)	1
Plasmid (pOCC102-eIF4G or pOCC102-Pab1) (0.1 μg/μL)	1
  b. Add transfection mix to a 24-well plate together with 200 μL of 5 × 10^6^ Sf9 cells/mL stock (this will result in 1 × 10^6^ cells/mL). Determine cell density with Countess Cell Counting Chamber Slides and a Countess II Automated Cell Counter.
  c. Cover plate with a Breather-Easy membrane.
  d. Seal membrane and plate with parafilm.
  e. Place sealed plate at 27°C with shaking at 200 rpm for ∼16 h.
  f. Next day, add 1 mL of 4% PBS with 2% Pen-Strep (this will result in ∼2% FPS with ∼1% Pen-Strep).
  g. Place sealed plate at 27°C with shaking at 200 rpm for 3 days.
  h. Collect cells at 500 × *g* for 5 min at ∼23°C.
  i. Collect 1.5 mL of supernatant. This contains the released P1 virus.
  j. To generate P2 virus, add 50 μL of P1 virus to 50 mL of 0.5 × 10^6^ Sf9 cells.
  k. Add FBS and Penicillin-Streptomycin to a final concentration of 2% and 1% respectively.
  l. Place at 27°C with shaking at 200 rpm for 5 days.
  m. Collect cells at 2,000 × *g* for 5 min at ∼23°C.
  n. Collect the supernatant. This contains the released P2 virus.
  o. Store P2 virus at 4°C and protected from light.
    ***Note:*** P2 virus is good for ∼6 months when stored at 4°C.
2. Express MBP-eIF4G and MBP-Pab1p in Sf9 cells.
  a. Prepare 1 L of Sf9 cells in ESF AF Insect Cell Culture Medium at a cell density of 2 million cells/mL.
  b. Infect cells with 10 mL of P2 baculovirus containing recombinant DNA for the expression of MBP-eIF4G or MBP-Pab1p (step 1n).
  c. Incubate infected Sf9 cells at 27°C with shaking at 100 rpm for 65 h.

***Note:*** For optimal expression, we recommend monitoring the expression of MBP-eIF4G and MBP-Pab1p at different time points following infection (see troubleshooting problem 1).

3. Harvest and lyse cells.
  a. Harvest cells by centrifugation at 210 × *g* for 10 min at ∼23°C.
  b. Add one cOmplete protease inhibitor cocktail tablet and 0.1 U/mL benzonase to 100 mL Buffer A and place on ice.
  c. Resuspend cell pellet with 100 mL of ice-cold Buffer A supplemented with a cOmplete protease inhibitor cocktail tablet and 0.1 U/mL benzonase (step 3b).
    ***Note:*** Use buffer with high salt concentrations at all purification steps (>500 mM KCl), especially for the purification of eIF4G. The high salt concentration dissociates eIF4G-RNA complexes and represses eIF4G condensate assembly.
  d. Lyse cells with a LM10 pneumatic microfluidizer processor cooled with ice.
    i. Lyse cells with a pressure of 5,000 pound-force per square inch (psi).
  e. Clear the lysate with centrifugation at 75,600 × *g* for 30 min at 4°C using rotor JA 25.50 and Beckman Coulter Avanti J-25 High Speed Centrifuge.
4. Affinity capture of MBP-eIF4G or MBP-Pab1p.
  a. Transfer the supernatant of the cleared lysate to 5 mL of amylose resin equilibrated with Buffer A.
  b. Incubate for 1 h at ∼23°C with 10 rations/min.
  c. Place resin into an empty 20 mL Econo-Pac Chromatography Column.
  d. Wash resin with 10 column volumes (CV) of Buffer A.
  e. Add 4 CV of buffer A supplemented with 20 mM maltose to the resin and collected the elution containing MBP-eIF4G or MBP-Pab1p.
  f. Measure protein concentration in the elution with absorbance at 280 nm (A_280_)
    i. Extinction coefficients of MBP-eIF4G and MBP-Pab1p are 139,370 and 106,245 M^−1^cm^−1^ respectively.

***Note:*** Typical yield of MBP-eIF4G and MBP-Pab1p at this step from 1 L of cells is ∼5 mg and ∼10 mg, respectively.

5. Cleave MBP.
  a. Add HRV 3C protease to eluted proteins to a final protease:protein mass ratio of 1:100.
  b. Incubate for at least 1 h at ∼23°C.
6. Size exclusion chromatography (SEC) of eIF4G and Pab1p.
  a. Concentrate cleaved protein samples to 4 mL using Amicon Ultra Centrifugal Filters with 30 kDa molecular weight cutoff. Centrifuge at 4,000 × *g* for 15–20 min at 20°C.
  b. Filter protein samples with 0.22 μm PES syringe filter to remove aggregates.
  c. Load protein samples onto a HiLoad 16/600 Superdex 200 pg column equilibrated in Buffer B using an ÄKTA Pure Protein Purification System with a multi-wavelength UV-Vis monitor (measure A_280_ and A_254_).
  d. Fractionate the elution in 1.5 mL fractions.
  e. Check fractions with SDS-PAGE using 10% acrylamide gels (Figures 1C and 1D).
  f. Pool fractions containing eIF4G or Pab1p (Figures 1E and 1F).
    i. eIF4G retention volume: ∼65 mL
    ii. Pab1p retention volume: ∼75 mL

**CRITICAL:** Do not pool fractions with eIF4G or Pab1p that elute close to the void volume of the column. While these fractions contain eIF4G and Pab1, they also contain a high contamination of nucleic acids and irreversibly aggregated proteins (Figures 1E and 1F). Pooling fractions with aggregated and/or nucleic acid contamination will affect downstream experiments, such as the assembly of mRNPs and condensates.

7. Store purified eIF4G and Pab1p.
  a. Measure protein concentrations in pooled fractions with A_280_ and concentrate proteins with Amicon Ultra Centrifugal Filters with 30 kDa molecular weight cutoff to a final concentration of ∼80 μM for eIF4G and ∼200 μM for Pab1p. Centrifuge at 4,000 × *g* at 20°C.
    i. Extinction coefficients of eIF4G and Pab1p are 73,020 M^−1^cm^−1^ and 39,895 M^−1^cm^−1^ respectively.
      ***Note:*** The final yield of purified eIF4G and Pab1p from 1 L of cells is ∼2 mg and ∼4 mg, respectively and have a 260/280 nm ratio of ∼0.5–0.6, indicating little nucleic acid contamination.
  b. Store proteins in 5–10 μL aliquots in PCR tubes.
  c. Flash-freeze in liquid nitrogen and store at −80°C.

### Purification of eIF4E

**Timing: 3 days**

**Figure 2:**
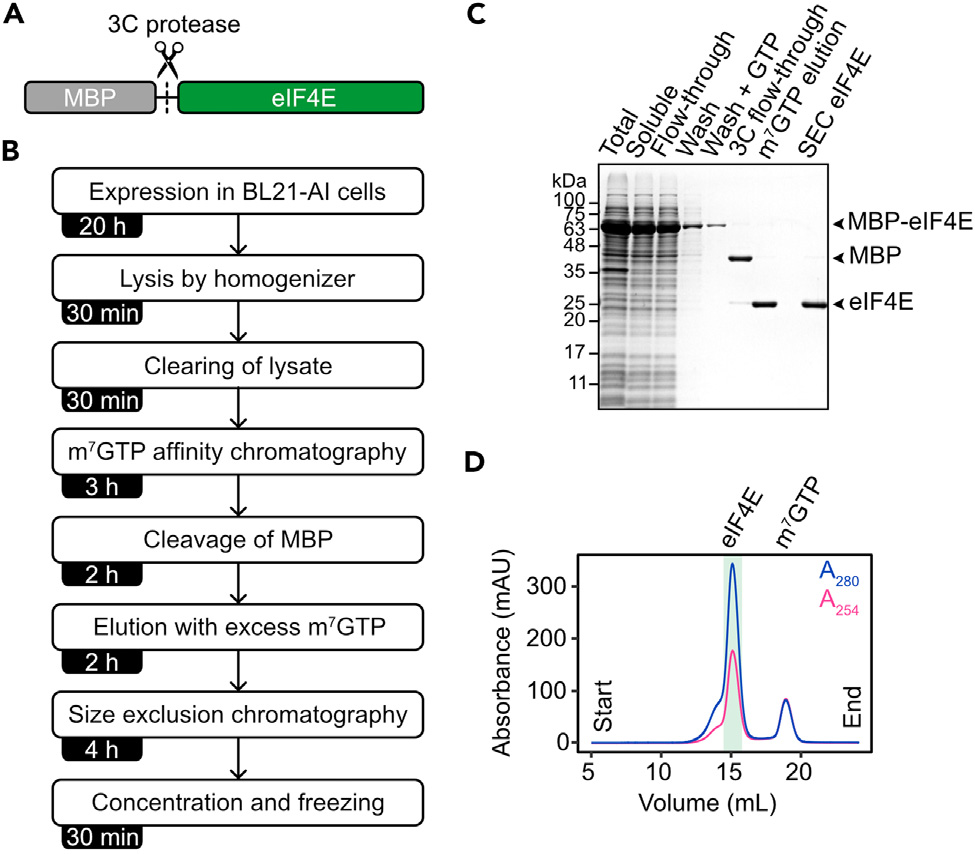
Purification of eIF4E (A) Schematic of MBP-eIF4E fusion protein construct. The MBP is cleaved with HRV 3C protease. (B) Steps for the purification of eIF4E. (C) Coomassie stained 15% acrylamide gel of the purification steps of eIF4E. (D) SEC chromatogram of eIF4E purification. A_280_ and A_254_ are shown. The elution peak with purified eIF4E is indicated.

eIF4E is recombinantly expressed and purified from chemically competent BL21-AI *E. coli* cells (Figures 2A and 2B). eIF4E is expressed as a fusion construct with maltose binding protein (MBP) placed at the N-terminus. In this case, MBP is not utilized for affinity capture on amylose resin but to increase the solubility of eIF4E and improve the yield of purified protein. MBP-eIF4E is captured on resin covalently modified with m^7^GTP prior to SEC (Figure 2C). To prevent the purification of other cap-binding proteins, we recommend purifying eIF4E from *E. coli*.

8. Express eIF4E in BL21-AI cells.
  a. Transform BL21-AI chemically competent cells with plasmid encoding MBP-eIF4E.
    i. Select cells by plating on LB agar plate with 50 μg/mL kanamycin.
      **CRITICAL:** Make sure that the yeast coding sequence of eIF4E is codon optimized for expression *E. coli*. Utilize online tools, such as the Codon Optimization Tool of IDT and chose *Escherichia coli K-12* for codon optimization of your sequence. If not codon optimized, the protein is prone to degradation when expressed in BL21-AI *E. coli* due to codon bias and the synthesis of incomplete or misfolded proteins.
  b. Inoculate a single colony into 20 mL of LB with 50 μg/mL kanamycin and place at 37°C with shaking at 180 rpm for ∼16 h.
  c. Next day, dilute the culture in 500 mL of LB with 50 μg/mL kanamycin to an optical density or absorbance at 600 nm (A_600_) of 0.05.
  d. Place at 37°C and grow to an optical density A_600_ of 0.5–0.6 with shaking at 180 rpm.
  e. Cool down the culture by placing it at 20°C with shaking at 180 rpm for 30 min.
  f. Induce protein expression with 0.1 mM IPTG and 0.2% L-arabinose.
    ***Note:*** The expression of T7 RNA polymerase in Bl21 AI cells is under control of *araBAD* promoter and induced with the addition of L-arabinose. This allows for tight control of gene expression and represses leaky expression of the protein of interest.
  g. Place the culture at 20°C with shaking at 180 rpm for ∼16 h.
9. Harvest and lyse cells.
  a. Harvest cells by centrifugation at 4,000 × *g* for 10 min at ∼23°C.
  b. Add one cOmplete protease inhibitor cocktail tablet and 0.1 U/mL benzonase to 100 mL Buffer E and place on ice.
  c. Resuspend cell pellet with 100 mL of cold Buffer E supplemented with a cOmplete protease inhibitor cocktail tablet and 0.1 U/mL benzonase (step 9b).
    ***Note:*** Use buffer with low salt concentrations at all purification steps, because eIF4E can irreversibly aggregate at high salt concentrations (>500 mM KCl).
  d. Lyse cells with a LM10 pneumatic microfluidizer processor cooled with ice.
    i. Lyse cells with a pressure of 12,000 pound-force per square inch (psi).
    ii. Repeat the previous step for complete lysis of cells.
  e. Clear the lysate with centrifugation at 75,600 × *g* for 30 min at 4°C using rotor JA 25.50 and Beckman Coulter Avanti J-25 High Speed Centrifuge.
10. Affinity capture of MBP-eIF4E.
  a. Transfer the supernatant of the cleared lysate to 3 mL of Immobilized γ-Aminophenyl-7- Methyl-guanosine-5′-triphosphate (m^7^GTP) (C10-spacer) resin equilibrated in Buffer E.
  b. Incubate for 1 h at 4°C with 10 rotations/min.
    **CRITICAL:** We strongly recommend using m^7^GTP resin for the capture of eIF4E. When not bound to m^7^GTP, eIF4E is prone to irreversibly aggregate at high concentrations (see troubleshooting problem 2). Affinity capture with amylose or nickel resin will result in the irreversible aggregation of eIF4E due to the high concentration of captured eIF4E on the resin. Alternatively, eIF4E can be captured with amylose or nickel resin, but m^7^GTP sodium salt should be added to the buffer to stabilize eIF4E.
  c. Place resin into an empty 20 mL Econo-Pac Chromatography Column.
  d. Wash resin with 10 column volumes (CV) of Buffer E.
  e. Wash resin with 10 column volumes (CV) of Buffer E supplemented with 100 μM GTP to remove non-specific binders from the resin.
11. Cleave MBP.
  a. Incubate the resin with Buffer E supplemented with 0.01 mg/mL of HRV 3C protease for 1 h at ∼23°C to cleave MBP.
  b. Wash resin with 10 CV of buffer E to remove cleaved MBP.
  c. Elute eIF4E from the resin with 4 CV of Buffer E supplemented with 100 μM m^7^GTP sodium salt.
  d. Measure protein concentration in elution.

***Note:*** m^7^GTP sodium salt absorbs at 280 nm. Measure protein concentrations using colorimetric methods, such as with the Bio-Rad Protein Assay.

***Note:*** Typical yield of eIF4E at this step is ∼3.5 mg from 500 mL of cells.

12. SEC of eIF4E.
  a. Concentrate elution to ∼400 μL using Amicon Ultra Centrifugal Filters with 3 kDa molecular weight cutoff. Centrifuge at 4,000 × *g* for ∼20 min at 20°C.
  b. Filter eIF4E elution with 0.22 μm PES syringe filter to remove aggregates.
  c. Load eIF4E elution onto a Superdex 200 Increase 10/300 GL column equilibrated in Buffer E using an ÄKTA Pure Protein Purification System with a multi-wavelength UV-Vis monitor (measure A_280_ and A_254_).
  d. Fractionate the elution in 0.2 mL fractions.
  e. Check fractions with SDS-PAGE using 15% acrylamide gels (Figure 2C).
  f. Pool fractions containing eIF4E (Figure 2D).
    i. eIF4E elution volume: ∼15 mL

***Note:*** If too concentrated, a fraction of eIF4E may be aggregated but can be separated by SEC. The aggregated fraction appears as a left-shoulder in the chromatogram (Figure 2D). If this is the case, take fractions that exclude this shoulder.

13. Store purified eIF4E.
  a. eIF4E can be flash-frozen in liquid nitrogen and stored at −80°C directly after SEC.
    i. Typically, the concentration of eIF4E in elution fractions from SEC is ∼50 μM.
  b. Alternatively, eIF4E can be concentrated to 100 μM with 100 μM m^7^GTP sodium salt with Amicon Ultra Centrifugal Filters with 3 kDa molecular weight cutoff prior to freezing and storage. Centrifuge at 4,000 × *g* at 20°C for 5 min. Monitor protein concentration using colorimetric methods, such as with the Bio-Rad Protein Assay.
    ***Note:*** The final yield of purified eIF4E from 500 mL of cells is ∼3 mg.
  c. Store proteins in 5–10 μL aliquots in PCR tubes.
  d. Flash-freeze in liquid nitrogen and store at −80°C.

### *In vitro* transcription of mRNA

**Timing: 2 days**

mRNA encoding Nanoluciferase (NLuc) with sequence specific 5′ and 3′ UTRs is synthesized with commercially available *in vitro* transcription kits. The synthesized mRNA contains a 5′ cap and a poly(A) tail.

**Figure 3:**
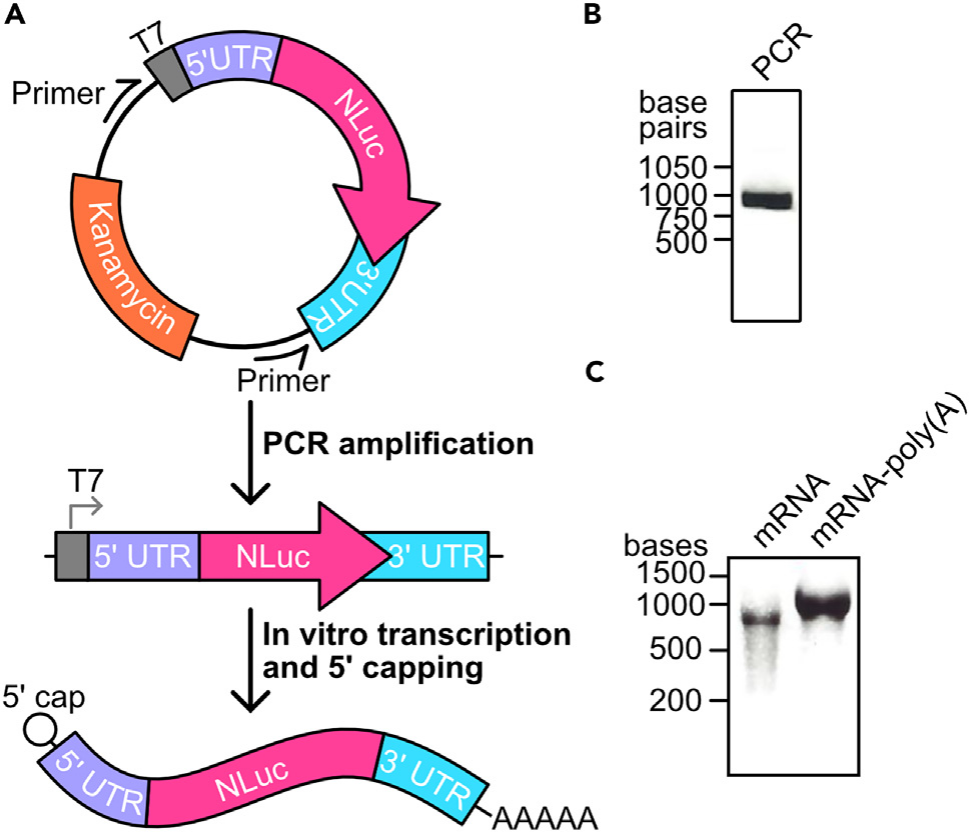
*In vitro* transcription of mRNA (A) Schematic of the steps for the synthesis of capped and polyadenylated *NLuc* mRNA. (B) DNA gel of PAB1-NLuc PCR product. (C) RNA gel of *in vitro* transcribed *PAB1-NLuc* mRNA before and after polyadenylation.
    **Table:** Preparation of PCR reaction mixture

14. Amplify DNA template from plasmid encoding Nanoluciferase (NLuc) using PCR.
  a. Amplify the DNA using primers that bind directly upstream of the T7 promoter and downstream of the 3′UTR sequence (Figure 3A).
    ***Note:*** Plasmid utilized in Desroches Altamirano et al., 2024^1^ (pUC57-T7-PAB1-NLuc) contains the 5′UTR of *PAB1* mRNA and a random 3′UTR sequence. The 5′UTR and 3′UTR sequences can be replaced with any other sequence of interest. To synthesize *PAB1-NLuc* mRNA, use primers CDA1 and CDA2 to amplify DNA from pUC57-T7-PAB1-NLuc and use the PCR product for mRNA synthesis.

Reagent	Volume (μL)
Nuclease free water	33.25
5× Phusion HF Reaction Buffer	10
10 mM dNTPs	1
10 μM Forward Primer (CDA1)	2.5
10 μM Reverse Primer (CDA2)	2.5
100 ng/μL plasmid (pUC57-T7-PAB1-NLuc)	0.25
Phusion High-Fidelity DNA Polymerase	0.5

**Table:** PCR steps

Step	Temperature (°C)	Time	# Cycles
Initial denaturation	98	1 min	
Denaturation	98	15 s	35
Primer annealing	55	20 s	
Extension	72	40 s	
Final extension	72	5 min
  b. Check PCR product with a 1% agarose gel. The size of The PCR product is 830 bp (Figure 3B).
  c. Purify the PCR product using a Monarch PCR & DNA purification kit.
    ***Note:*** Other DNA and PCR clean-up kits can be used.
15. Synthesize mRNA using mMESSAGE mMACHINE T7 Transcription Kit from Thermo Fisher Scientific.
  ***Note:*** Please refer to the manufacturer’s protocol (mMESSAGE mMACHINE Kit). Alternative kits can be used. However, make sure that the kit contains mRNA capping enzymes or that capping enzymes are supplemented.
  a. Combine reagents in the following order at ∼23°C:
    **Table:** Preparation of an *in vitro* transcription reaction

Reagent	Volume (μL)
2× NTP/CAP	10
10× Reaction Buffer	2
Cleaned PCR template (0.3–0.5 μg total)	6
Enzyme mix	2
Optional: Aminoallyl-UTP-ATTO-488	1

    ***Note:*** Aminoallyl-UTP-ATTO-488 can be added to synthesize fluorescently labeled mRNA.
    **CRITICAL:** Make sure that 2× NTP/CAP and 10× Reaction buffer are thawed completely before use. Components in the 10× Reaction Buffer and 2× NTP/CAP can precipitate. Pipette up and down until no precipitates are observed. If not properly mixed, the yield of transcribed mRNA may be low (see troubleshooting problem 3).
  b. Mix the reaction by carefully pipetting up and down.
  c. Place at 37°C for 2 h.
  d. Add 2 μL of TURBO DNase and place at 37°C for 15 min to digest the PCR template.
16. Polyadenylate mRNA with Poly(A)-Tailing Kit from Thermo Fisher Scientific.
  ***Note:*** Please refer to the manufacturer’s protocol (Poly(A) Tailing Kit). Alternative kits can be used.
  a. Combine reagents in the following order at ∼23°C:
    **Table:** Preparation of polyadenylation reaction

Reagent	Volume (μL)
*In vitro* transcribed mRNA	20
Nuclease free water	36
5× E-PAP Buffer	20
25 mM MnCl_2_	10
10 mM ATP	10
E-PAP	4
  b. Mix reaction by carefully pipetting up and down.
  c. Place at 37°C for 30 min.
    ***Note:*** A 30 min incubation results in the addition of 100–200 A nucleotides to mRNA (Figure 3C). For longer poly(A) tails, increase the incubation time.
17. Precipitate poly(A) tailed mRNA.
  a. Add 100 μL of 7.5 M lithium chloride.
  b. Place at −20°C for at least 1 h to ∼16 h to precipitate the mRNA.
  c. Centrifuge at 21,000 × *g* for 20 min at 4°C.
  d. Wash pellet with 1 mL of cold 70% ethanol.
  e. Centrifuge at 21,000 × *g* for 10 min at 4°C.
  f. Remove as much liquid as possible and dry the pellet at ∼23°C for 15–20 min until the pellet becomes translucent.
  g. Resuspend pellet in 100 μL of nuclease-free water.
  h. Centrifuge at 21,000 × *g* for 5 min at ∼23°C to remove any mRNA aggregates.
  i. Transfer the supernatant to a new tube.
  j. Measure mRNA concentration.
    i. Typical concentrations of mRNA are ∼1,000–1,500 ng/μL or 2–3 μM.
  k. Store mRNA in 5–10 μL aliquots in PCR tubes.
  l. Flash-freeze in liquid nitrogen and store at −80°C.

***Note:*** mRNA can be thawed and re-frozen. However, limit freeze and thawing to three times because the mRNA may start to aggregate (see troubleshooting step 5).

18. Visualize synthesized mRNA using an RNA gel.
  a. Combine 2 μL of synthesized mRNA with 2 μL of 2× RNA loading dye.
  b. Heat at 95°C for 2 min.
  c. Place directly on ice.
  d. Load 4 μL of mRNA in 2% agarose gel in 1× TBE.
  e. Load 2 μL of RiboRuler High Range RNA Ladder in neighboring well.
  f. Run gel at 200 V for 10 min.
  g. Visualize mRNA with UV light (Figure 3C).

### Preparation of yeast extracts

**Timing: 2 days**

**Figure 4:**
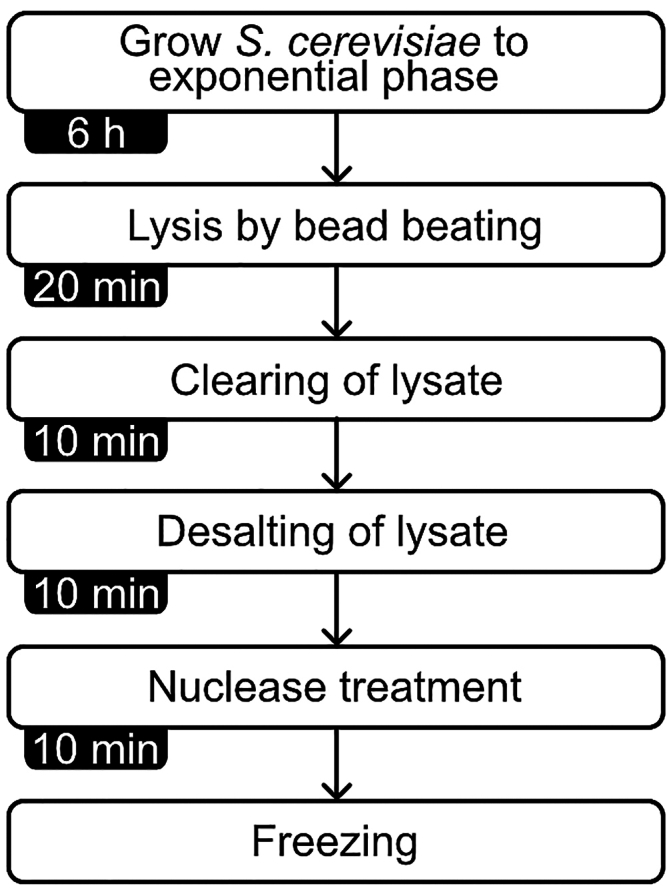
Steps for the preparation of cell-free yeast extracts

Cell-free extracts for *in vitro* translation assays are prepared from exponentially growing *S. cerevisiae* cells (Figure 4). Here, we outline a protocol that was adapted from Wu and Sachs 2014,^3^ starting with a 1 L culture of yeast cells and yielding ∼5 mL of yeast extract.

19. Grow S*. cerevisiae* cells to exponential phase.
  a. Inoculate *S. cerevisiae* strain W303 in 20 mL of YPD and place at 30°C with shaking at 200 rpm for ∼16 h.
    ***Note:*** Other strains of *S. cerevisiae* can be used to prepare extracts. In addition, genetically modified strains (with gene deletions or expressing fluorescently labelled proteins) can be used.
  b. Next day, dilute cells to an optical density A_600_ of 0.2 in 1 L of YPD and place at 30°C with shaking at 200 rpm for ∼6 h or until the culture reaches an optical density A_600_ of 1.0–1.2.
20. Harvest and lyse cells.
  ***Note:*** All following steps should be done on ice or at 4°C. We recommend that all following steps are done in succession.
  a. Harvest cells by centrifugation at 3,500 × *g* for 5 min at 4°C.
  b. Resuspend cell pellet with 30 mL of cold Buffer Y/M.
  c. Harvest cells by centrifugation at 2,000 × *g* for 4 min at 4°C.
  d. Wash cell pellet with 30 mL of cold Buffer Y/M.
  e. Repeat steps 20c-d three more times.
  f. On the last spin, centrifuge cells at 3,000 × *g* for 4 min at 4°C.
  g. Remove the supernatant and weigh the cell pellet.
    i. Typical weight of cell pellet from 1 L of cells is ∼3 g.
  h. Add one tablet of EDTA-free cOmplete protease inhibitor to 40 mL of Buffer Y/M and place on ice.
  i. Resuspend the cell pellet with 1.5 mL of cold Buffer Y/M supplemented EDTA-free cOmplete protease inhibitors per gram of cell weight.
    i. For a cell pellet of 3 g, resuspend the pellet in cold 4.5 mL of Buffer Y/M supplemented EDTA-free cOmplete protease inhibitors.
      **CRITICAL:** Make sure to use EDTA-free protease inhibitors because EDTA dissociates ribosomes and can inhibit the activity of enzymes in the extract.
  j. Combine 800 μL of resuspended cells with cold 500 μL of acid-washed glass beads (425–600 μm) in pre-chilled 1.5 mL tubes. Repeat this step for the remainder of resuspended cells.
  k. Lyse cells by beat beating using a Mixer Mill MM 400 set to 25 oscillations per second for 20 min at 4°C.
  l. Centrifuge tubes at 650 × *g* for 2 min to remove whole cells and reduce foam.
  m. Transfer the supernatant to pre-chilled centrifuge tubes and centrifuge at 21,000 × *g* for 10 min at 4°C to remove cellular debris.
  n. Transfer the supernatant to a Zeba spin desalting column with a 7 kDa molecular weight cut-off equilibrated with cold Buffer Y.
  o. Centrifuge at 215 × *g* for 3 min at 4°C.
21. Nuclease treatment and storage.
  a. To the flowthrough, add 2,000 gel units of micrococcal nuclease per mL of yeast extract and 1 mM CaCl_2_. Place at ∼23°C for 10 min for the digestion of nucleic acids.
  b. Stop micrococcal nuclease activity with the addition of 2.5 mM EGTA.
  c. Store yeast extract in 100–400 μL aliquots in 1.5 mL tubes.
  d. Flash-freeze with liquid nitrogen and store at −80°C.

### Reconstitution of mRNPs and condensates

**Timing: 30 min**

**Figure 5:**
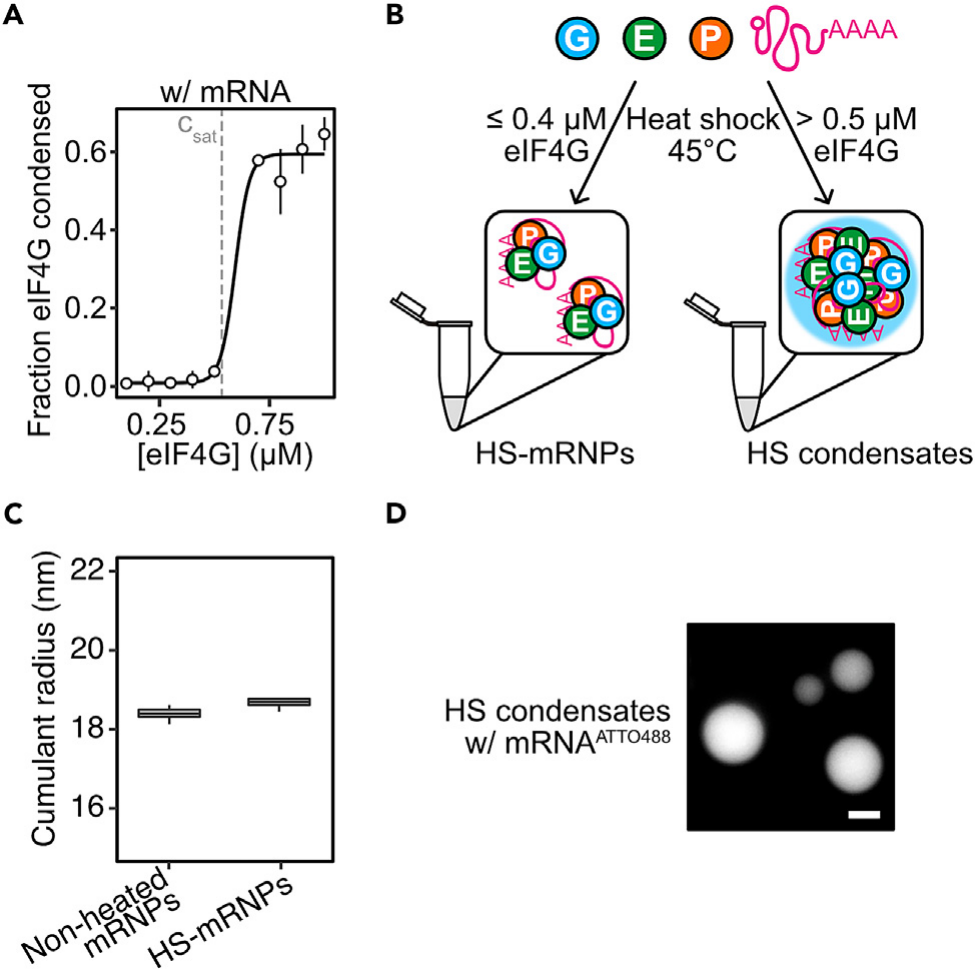
Reconstitution of HS-mRNPs and condensates (A) Fraction condensed of eIF4G with 70 nM *PAB1-NLuc* mRNA as a function of eIF4G concentration after exposure to 45°C for 10 min (mean, SD, fit, *n* = 4). Data adapted from Desroches Altamirano et al., 2024.^1^ (B) Schematic for the reconstitution of HS-mRNPs and condensates with purified eIF4G, eIF4E, Pab1p and synthesized *PAB1-NLuc* mRNA. (C) Representative dataset of DLS measurements of reconstituted non-heated or HS eIF4G-eIF4E-Pab1p-*PAB1-NLuc*-mRNPs. (D) Reconstituted condensates with 3.75 μM eIF4G, eIF4E, Pab1p and 150 nM *PAB1-NLuc* mRNA^ATTO488^ after heating at 45°C for 10 min. Scale, 5 μm.

In this step, we outline the procedure to reconstitute mRNPs and condensates. For comparative analyses in *in vitro* translation assays in step 28, we describe the reconstitution of non-heated and heated mRNPs and condensates. mRNPs and condensates are reconstituted using the same procedure, but with different protein concentrations. For the reconstitution of mRNPs, use eIF4G concentrations below the saturation concentration (c_sat_) (≤ 400 nM). For the reconstitution of condensates, use eIF4G concentrations above c_sat_ (> 500 nM) (Figures 5A and 5B).

22. Thaw proteins and leave at ∼23°C.
23. Centrifuge thawed proteins and mRNA at 21,000 × *g* for 5 min to remove aggregates.
24. Re-measure protein and mRNA concentrations at A_280_ and A_260_, respectively, with appropriate extinction coefficients.
25. Reconstitute non-heated and HS-mRNPs.
  ***Note:*** Below, we describe an example procedure for the reconstitution of non-heated and HS-mRNPs. Different combinations of proteins, mRNA, buffers, and heating temperatures can be used for comparative analyses.
  a. In a 1.5 mL tube, combine eIF4G, eIF4E and Pab1p to a 2× concentration.
    ***Note:*** Use low-binding tubes to reduce the adsorption of proteins and mRNA to the walls of tubes.
    **Table:** Example pipetting scheme for a 2× eIF4G-eIF4E-Pab1p solution

Reagent	Stock concentration (μM)	2× concentration (μM)	Volume (μL)
Buffer G	-	-	88.12
eIF4G	90	0.8	0.80
eIF4E	100	0.8	0.72
Pab1p	200	0.8	0.36
  b. To a 1.5 mL tube, dilute mRNA to a 2× concentration.
    **Table:** Example pipetting scheme for a for a 2× mRNA solution

Reagent	Stock concentration (μM)	2× concentration (μM)	Volume (μL)
Buffer G	-	-	85.5
NLuc mRNA	2.8	0.14	4.5
  c. In a 1.5 mL tube, combine protein and mRNA solutions in a 1:1 volume ratio.
  d. For the reconstitution of HS-mRNPs, place the protein-mRNA sample in a ThermoMixer for 10 min at 45°C.
    ***Note:*** We recommend that additional analyses are utilized to characterize the reconstituted mRNPs. For example, we recommend measuring the size distribution of particles using dynamic light scattering (DLS).
  e. Confirm the reconstitution of mRNPs with DLS using the Prometheus Panta.
    i. Load 10 μL of sample into high-sensitivity capillaries.
    ii. Perform a high sensitivity size analysis at 25°C.
    iii. Analyze and plot cumulant radius measurements (Figure 5C).
26. Reconstitute condensates.
  ***Note:*** Below, we describe an example procedure for the reconstitution of condensates heated at different temperatures. In this case, we prepare protein-mRNA samples at a five-fold higher concentration, so that the final concentration of proteins in *in vitro* translation (IVT) assays in step 28 is 750 nM. Different combinations of proteins, mRNA, buffers and heating temperatures can be used for comparative analyses.
  a. In a 1.5 mL tube, combine eIF4G, eIF4E, Pab1p and mRNA at a 5× concentration.
    **Table:** Example pipetting scheme for a 5× eIF4G-eIF4E-Pab1p-mRNA solution

Protein	Stock concentration (μM)	5× dilution concentration (μM)	Volume (μL)
Buffer G	-	-	42.6
eIF4G	90	3.75	2.08
eIF4E	100	3.75	1.88
Pab1p	200	3.75	0.94
mRNA	2.8	0.14	2.5

    ***Note:*** We recommend the use of low-binding tubes to reduce the adsorption of proteins and mRNA to the walls of tubes.
  b. Aliquot 5× eIF4G-eIF4E-Pab1p-mRNA solution in 8 μL in 1.5 mL low-binding tubes and place at 30°C, 37°C, 40°C, 42°C or 45°C for 10 min using a ThermoMixer.
    ***Note:*** We recommend imaging the samples for the presence of condensates (Figure 5D). In this case, we recommend utilizing fluorescently labelled mRNA (step 15a) or fluorescently labelled proteins, as shown in Desroches Altamirano et al., 2024.^1^ If you encounter difficulties in reconstituting condensates, refer to troubleshooting problem 4.
    **CRITICAL:** Condensates settle with time and can adhere to the plastic wall of tubes, therefore subsequent experiments (such as in IVT assays) should be done directly after.

### *In vitro* translation assay

**Timing: 2 h**

**Figure 6:**
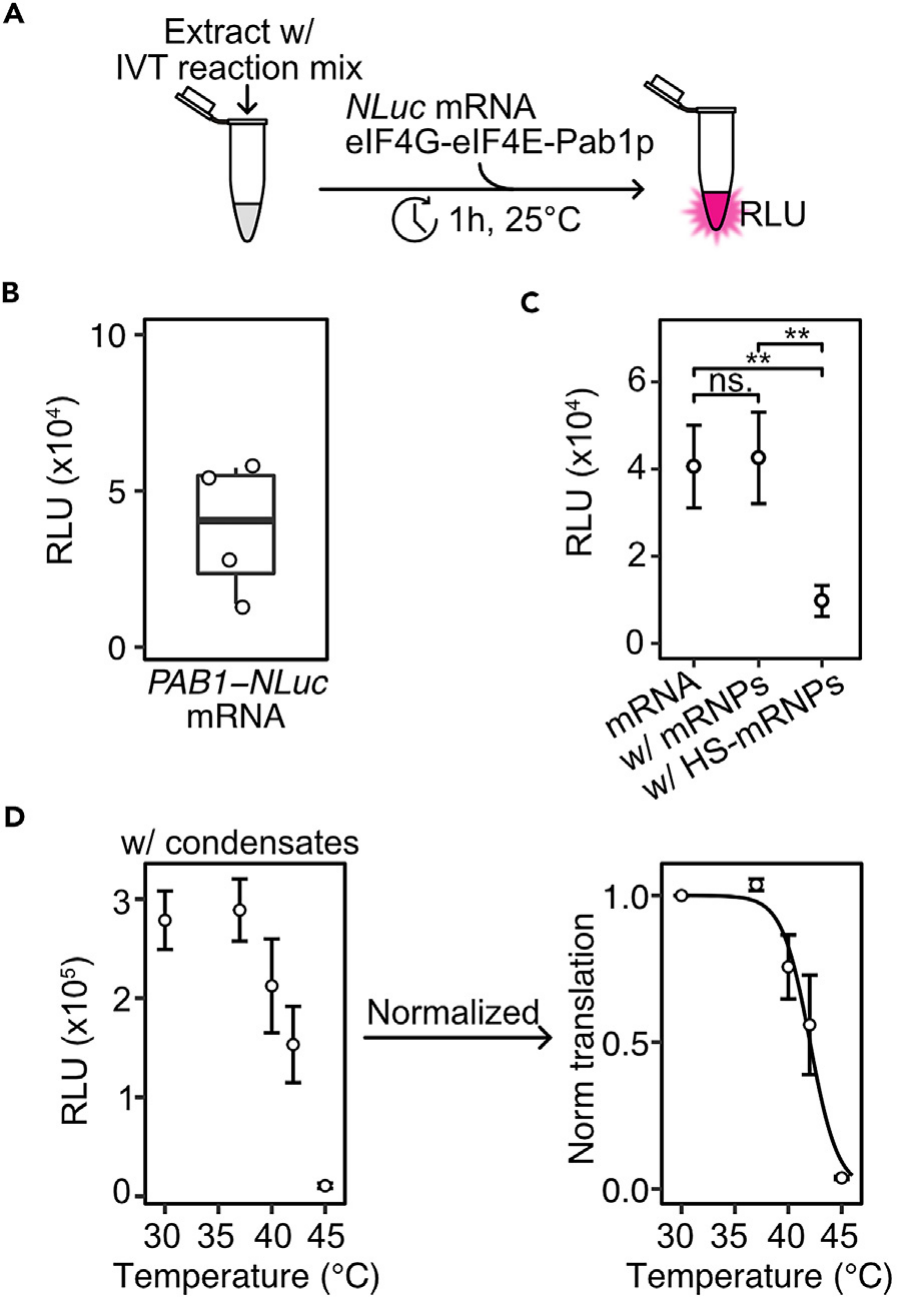
*In vitro* translation (IVT) assay with reconstituted mRNPs and condensates (A) Schematic of *in vitro* translation assay (IVT). (B) Translation of *PAB1-NLuc* mRNA in cell-free yeast extracts. Relative luminescence units (RLU) measurements from four independent extract preparations are plotted. (C) RLU following the addition of non-heated (25°C) or heated (45°C) eIF4G-eIF4E-Pab1p-*PAB1-NLuc* mRNPs to yeast extracts (mean, SD, *n* = 3). Significance levels: p-values ∗ < 0.05, ∗∗ < 0.01, ∗∗∗ < 0.001. Data adapted from Desroches Altamirano et al., 2024.^1^ (D) Raw RLU (left) and normalized fold change in translation (right) following addition of eIF4F-Pab1p and *PAB1-NLuc* mRNA condensates pre-heated at the indicated temperatures for 30 min (mean, SD, fit, *n* = 3). Data adapted from Desroches Altamirano et al., 2024.^1^

In this step, we outline the protocol for *in vitro* translation (IVT) assays using yeast extracts and reconstituted mRNPs and condensates. The mRNPs and condensates reconstituted in step 25 and step 26 are added to nuclease-treated yeast extracts prepared in step 21 supplemented with *in vitro* translation reaction mix (Figure 6A). The translation of *NLuc* mRNA associated with mRNPs or condensates is determined by measuring relative luminescence units (RLU) using a Spark multimode microplate reader.

27. Prepare IVT reaction mix.
  a. See materials and equipment for details.
28. Prepare *in vitro* translation assay.
  a. Thaw nuclease-treated extract prepared in step 21 on ice.
  b. Place IVT reaction mix on ice.
  c. Combine nuclease-treated extract with IVT reaction mix in a 1:2 volume ratio and place on ice.
    ***Note:*** One IVT reaction requires a combined volume of 12 μL of yeast extract + IVT reaction mix (see table below). For multiple samples, prepare a master mix by upscaling the volumes of extract and IVT reaction mixture.
    **Table:** Preparation of nuclease-treated yeast extract + IVT reaction mix

Component	Volume (μL)
Nuclease-treated extract (step 21)	8
IVT reaction mix	4
Total	12
  d. Add 3 μL of reconstituted mRNPs (prepared in step 25) or 3 μL of condensates (prepared in step 26) to nuclease-treated extract + IVT reaction mix.
    ***Note:*** Samples can be prepared in 1.5 mL tubes, PCR tubes or in imaging plates. When possible, low-binding tubes or plates (such as CellCarrier Ultra ULA-Coated 384-well Microplate) should be used to minimize protein and/or mRNA adsorption to the plastic.
    **Table:** Example of experimental set-up for IVT assay with reconstituted mRNPs

Sample	Vol total (μL)	Proteins/mRNA (μL)	Extract-IVT reaction mix (μL)
mRNA alone	15	3	12
Non-heated mRNP (step 25c)	15	3	12
HS-mRNP (step 25d)	15	3	12

**Table:** Example of experimental set-up for IVT assay with reconstituted condensates

Sample	Pre-incubation temperature (°C)	Vol total (μL)	Proteins/mRNA (μL)	Extract-IVT reaction mix (μL)
Condensates (step 26b)	30	15	3	12
	37	15	3	12
	40	15	3	12
	42	15	3	12
	45	15	3	12
  e. Mix samples by flicking the tube or by gently pipetting up and down.
  f. Place at 25°C for 60 min.
  g. Flash-freeze in liquid nitrogen and store at −80°C.
29. Measure luminescence.
  ***Note:*** We recommend the Nano-Glo Luciferase Assay System to measure luminescence.
  a. Thaw IVT reactions on ice.
  b. Thaw the Nano-Glo Luciferase Assay buffer at ∼23°C.
  c. Prepare a mixture of Nano-Glo Luciferase Assay Buffer and Substrate to a final buffer:substrate volume ratio of 50:1.
  d. Add an equal volume of Nano-Glo Luciferase Assay Buffer-Substrate mixture to the IVT reactions.
  e. Transfer 5 μL of IVT reaction with Nano-Glo Luciferase Assay Buffer-Substrate mixture to a 384-well Low Volume White Round Bottom Polystyrene NBS Microplate.
    ***Note:*** Use white plates for luminescence measurements to maximize light output signal. To prevent crosstalk between adjacent wells, place samples in alternating wells.
  f. Measure luminescence using a Spark multimode microplate reader. If RLU measurements are low, refer to troubleshooting problem 5.
    i. Measure luminescence signal (counts) between 440-475 nm (maxima of light output) with a 1 s integration time.
    ii. If luminescence measurements exceed the detection limit of the detector, utilize attenuation filters or dilute the sample as needed.
  g. Clean the microplate with water and 20% ethanol and dry at ∼23°C. The plate can be re-used for future measurements.

## Expected outcomes

Examples of data from IVT assays is shown in Figures 6B–6D. Typically, relative luminescence unit (RLU) measurements in IVT assays with *PAB1-NLuc* mRNA are in the range of 2,000–5,000 counts/s (Figure 6B). When the mRNA is associated with HS-mRNPs or HS-condensates, the RLU decreases (Figures 6C and 6D), demonstrating that the mRNA is translationally repressed and inaccessible to the ribosomes in the extract. The raw data can be normalized to demonstrate the fold-change in translation (Figure 6D).

## Limitations

The assembly of translationally arrested HS-mRNPs and HS-condensates necessitates a strong association between the mRNA and translation factors. Additional experiments, including the monitoring of the apparent binding and dissociation of proteins to mRNA are thus necessary. As an example, refer to Desroches Altamirano et al., 2024.^1^

Cellular constituents may be depleted during the preparation of yeast extracts. We have noticed that in the absence of EDTA in the lysis buffer, some translation factors and ribosomal constituents are depleted from the supernatant after step 20m. Irrespective of this limitation, RLU measurements between extract preparations are reproducible (Figure 6B).

We would also like to emphasize that yeast extracts already contain the proteins supplemented in mRNPs and condensates (e.g., eIF4G, eIF4E and Pab1p). However, this protocol is utilized as a comparative study of the accessibility of mRNA associated in mRNPs and condensates reconstituted *in vitro*, as demonstrated in Desroches Altamirano et al., 2024.^1^ Alternatively, extracts prepared from cells with gene knockouts or depleted for proteins of interest can be utilized.

## Troubleshooting

### Problem 1

Yield of purified eIF4G is low.

### Potential solution

Increase the volume of insect cells to 2 L. Monitor the expression of MBP-eIF4G in insect cells over 3 days by SDS-PAGE to identify the optimal time point for maximal protein expression and cell harvest (step 2c). Do not express proteins for more than 3 days, because this can result in increased protein degradation and cell death.

The affinity capture of MBP-eIF4G with amylose resin is poor (Figure 1C) (step 4). Increasing the volume of resin does not lead to an increased capture of MBP-eIF4G, indicating that a fraction of MBP in expressed proteins is inaccessible for binding to amylose. We have tested different buffer compositions to improve affinity capture and concluded that buffer with high salt improves affinity capture and subsequent purification steps. Critically, SEC in buffer with lower salt (< 300 mM KCl) results in significant loss of eIF4G due to non-specific binding of eIF4G to the matrix of the column.

### Problem 2

eIF4E aggregates irreversibly.

### Potential solution

eIF4E irreversibly aggregates at high concentrations. This is already observed in cells where eIF4E is predominantly insoluble. Use of solubilizing tag, such as MBP, increases the yield of soluble eIF4E (Figure 2C). In addition, expression of eIF4E in bacteria at 18°C–20°C increases solubility. To prevent the aggregation of eIF4E during purification and at any concentration step, supplement the buffer with 100 μM m^7^GTP sodium salt. Do not concentrate eIF4E above the concentration of supplemented m^7^GTP sodium salt. If the concentration of eIF4E after affinity capture on m^7^GTP resin is too high, some eIF4E may aggregate. However, these aggregates can be separated by SEC (Figure 2D) (step 12).

### Problem 3

Yield of synthesized mRNA is low.

### Potential solution

Use purified PCR products for *in vitro* transcription reactions (step 14c). Make sure that there are no precipitates in the 10× Reaction Buffer or 2× NTP/CAP solutions (step 15a). Typically, a pellet should appear after lithium chloride precipitation (step 17c). If not, add more lithium chloride and place at −20°C for at least another 1 h before repeating step 17c.

### Problem 4

Difficulty in reconstituting condensates.

### Potential solution

The concentrations detailed in step 26 should result in the assembly of condensates (Figure 5D), irrespective of the incubation temperature. Given that condensates can adsorb to plastic surfaces, use low binding tubes when possible. In addition, when transferring condensates to an imaging plate, pipet gently because condensates (especially non-heated condensates) are susceptible to shear stress and dissolution by pipetting. In general, remove unnecessary transfer steps when dealing with condensates.

### Problem 5

Yeast extracts are poorly active and RLU measurements are low.

### Potential solution

Make sure that none of the buffers and reagents contain EDTA. Extracts prepared from cells that are not in exponential phase can be less active. Harvest cells that are exponentially growing in YPD with an optical density A_600_ 0.8–1.2 (step 19b). Low RLU values may also indicate reduced activity of CPK in the IVT reaction mix. Use IVT reaction mix that is less than 3 months old for optimal results. In addition, synthesized mRNA can aggregate when stored for longer than 6 months at −80°C. If aggregated, the concentration of “functional” mRNA is decreased in the IVT assay. Centrifuge mRNA at 21,000 × *g* for 5 min and re-measure the RNA concentration in the supernatant before each experiment.

## Resource availability

### Lead contact

Further information and requests for resources and reagents should be directed to and will be fulfilled by the lead contact, Prof. Dr. Simon Alberti (simon.alberti@tu-dresden.de).

### Technical contact

Any information regarding the details of the protocol should be directed to and will be fulfilled by the technical contact, Dr. Christine Desroches Altamirano (christine.desroches@tu-dresden.de).

### Materials availability

Plasmids utilized in this study are deposited in Addgene.

All unique/stable reagents generated in this study are available from the lead contact with a completed Materials Transfer Agreement.

### Data and code availability

All data are archived at the Biotechnology Center (BIOTEC), Center for Molecular and Cellular Bioengineering, Technische Universität Dresden. All data reported in this paper will be shared by the lead contact upon request. This paper does not report original code.
